# Editorial: Sudden cardiac death: mechanisms, risk and prevention

**DOI:** 10.3389/fcvm.2026.1983912

**Published:** 2026-09-11

**Authors:** Dominik S. Westphal, Sebastian Clauss

**Affiliations:** 1 Institute of Human Genetics, Landeskrankenhaus University Hospital, Paracelsus Medical Private University, Salzburg, Austria; 2 Institute of Human Genetics, TUM Klinikum Rechts der Isar, School of Medicine and Health, Technical University of Munich, Munich, Germany; 3 Klinik und Poliklinik für Innere Medizin I, TUM Klinikum Rechts der Isar, School of Medicine and Health, Technical University of Munich, Munich, Germany; 4 Department of Medicine I, University Hospital Munich, Campus Großhadern, Ludwig-Maximilians University Munich (LMU), Munich, Germany; 5 Interfaculty Center for Endocrine and Cardiovascular Disease Network Modelling and Clinical Transfer (ICONLMU), LMU Munich, Munich, Germany; 6 Institute of Surgical Research at the Walter-Brendel-Centre of Experimental Medicine, University Hospital, LMU Munich, Munich, Germany; 7 DZHK (German Centre for Cardiovascular Research), Partner Site Munich, Munich Heart Alliance (MHA), Munich, Germany

**Keywords:** arrhythmia, autopsy, cardiogenetics, prevention, sudden cardiac death

Sudden cardiac death (SCD) is a sudden, unexpected death from a cardiovascular disease which occurs within one hour of symptom onset (witnessed) or if the patient was seen to be alive within the last 24 h (non-witnessed) (1). The average annual incidence of SCD in the European Union was calculated to be 36.8–39.7 per 100,000 based on data from different registries with men being affected more often than women (2). Considering the causes of SCD, they strongly depend on the age of the affected individual: while chronic heart diseases, such as coronary artery disease or valvular heart disease, are the major causes in older patients, hereditary arrhythmia syndromes and cardiomyopathies cause more than half of the SCD cases in patients below the age of 50 (3). Despite significant advances made over the last decades, SCD remains a major medical, social, and economic burden due to its high morbidity and mortality, the impact of remaining relatives who are potentially also affected by the underlying disease, and the high economic costs of SCD (4). Early diagnosis of patients and—in case of inherited disease—identification of asymptomatic family members at risk, a comprehensive risk stratification, optimized treatment, and preventive strategies are important challenges of our healthcare systems (5, 6). To address this, a better etiological (7) and pathophysiological understanding (8–10) of SCD and novel, innovative treatment and prevention strategies for diseases leading to SCD are required (11–13).

In this Research Topic “Sudden Cardiac Death: Mechanisms, Risk and Prevention” the contributors shed light on different aspects of SCD. Sun et al. provided an epidemiologic view and studied the incidence of sudden infant death syndrome (SIDS) over the last 30 years on a global, regional and national level. They noticed a continuing decline, even during the COVID-19 pandemic, although with regional differences.

Han reviews mechanisms of sudden cardiac arrest in patients with congenital heart disease, comprehensively summarizes the embryological heart development, and discusses current and future treatment strategies. Using data from autopsies Salzillo et al. emphasize the importance of a multidisciplinary approach in order to diagnose and prevent SCD in the young. They demonstrate that combined with genetic testing, autopsies can help to develop specific preventive strategies. In the context of genetic testing, Paz-Cruz et al. report three different cases with inherited Long QT syndrome (LQTS), which illustrate the synergistic effects of ECG findings, genetic testing and family history on diagnostic accuracy and personalized patient management.

When it comes to physical activity, there is an increased burden of SCD in athletes (14). Zhang et al. review different aspects of cardiac arrest and SCD specifically during marathon running. They describe mechanisms of exercise-triggered events and discuss future steps in precision risk stratification. However, there are also external risk factors for SCD in competitive sports: the abuse of anabolic-androgenic steroids (AAS). Based on autopsies as well as histopathological, immunohistochemical, and toxicological findings, Di Fazio et al. conclude that AAS-induced hypertrophy and fibrosis contribute to the risk of SCD and that prevention strategies should be used in order to reduce substance abuse in athletes. Another study by Radaelli et al. uses data from the Friuli Venezia Giulia Sudden Cardiac Death Register in the Young and also a multidisciplinary approach to examine causes of SCD in the Veneto region. Although several pathogenic genetic variants could be detected, polysubstance use was identified in most positive cases.

Apart from the causes of SCD, identifying patients at risk is of utmost importance for developing precise prevention strategies. A reduced ejection fraction (EF) ≤ 35% is commonly used as a predictive value for life-threatening arrhythmias, but in non-ischemic cardiomyopathies such as dilated cardiomyopathy (DCM) its value as a risk parameter seems to be limited (15, 16). Bradel et al. review various other parameters, including tissue characteristics identified by magnetic resonance imaging and electrophysiologic testing, and risk models that might help to improve risk stratification in patients with DCM. Studying the plasma proteome, Dai et al. identified a 71-protein signature which is independently associated with the occurrence of SCD and could help to improve risk prediction. Since a prolonged QTc interval does not only occur in inherited LQTS, but also in drug-induced LQTS, Steinbrech et al. investigate the clinical use of the Tisdale-risk-score in patients receiving QTc-prolonging drugs. The Tisdale-risk-score was initially published in 2013, estimating the risk of QT interval prolongation in hospitalized patients (17). Steinbrech et al. examine patients under the treatment with azole antifungal and show that the Tisdale-risk-score has a high sensitivity for identifying patient at risk for QTc prolongation.

Last, while most of the contributors concentrate on cardiovascular diseases or cardiac-related risks for SCD, Truyen et al. provide an overview on the role of non-cardiovascular comorbidities in SCD risk prediction and show that SCD may not be seen as an isolated cardiac disease.

This Research Topic intends to promote a better understanding of various causes for SCD by looking at it from different angles. Collected articles provide epidemiological insights, emphasize the importance of a multidisciplinary approach for identifying underlying causes by combining autopsy, imaging, clinical phenotyping and genetic testing, and discuss different approaches for risk stratification.
